# Impaired Eye Region Search Accuracy in Children with Autistic Spectrum Disorders

**DOI:** 10.1371/journal.pone.0058167

**Published:** 2013-03-14

**Authors:** John R. Pruett, Sarah Hoertel, John N. Constantino, Angela LaMacchia Moll, Kelly McVey, Emma Squire, Eric Feczko, Daniel J. Povinelli, Steven E. Petersen

**Affiliations:** 1 Department of Psychiatry, Washington University School of Medicine, Saint Louis, Missouri, United States of America; 2 Department of Pediatrics, Washington University School of Medicine, Saint Louis, Missouri, United States of America; 3 Department of Neurology, Washington University School of Medicine, Saint Louis, Missouri, United States of America; 4 Department of Biology, University of Louisiana at Lafayette, Lafayette, Louisiana, United States of America; 5 Department of Radiology, Washington University School of Medicine, Saint Louis, Missouri, United States of America; 6 Department of Psychology, Washington University, Saint Louis, Missouri, United States of America; 7 Department of Anatomy and Neurobiology, Washington University School of Medicine, Saint Louis, Missouri, United States of America; 8 Department of Biomedical Engineering, Washington University, Saint Louis, Missouri, United States of America; Ecole Normale Supérieure, France

## Abstract

To explore mechanisms underlying reduced fixation of eyes in autism, children with Autistic Spectrum Disorders (ASD) and typically developing children were tested in five visual search experiments: simple color feature; color-shape conjunction; face in non-face objects; mouth region; and eye region. No group differences were found for reaction time profile shapes in any of the five experiments, suggesting intact basic search mechanics in children with ASD. Contrary to early reports in the literature, but consistent with other more recent findings, we observed no superiority for conjunction search in children with ASD. Importantly, children with ASD did show reduced accuracy for eye region search (p = .005), suggesting that eyes contribute less to high-level face representations in ASD or that there is an eye region-specific disruption to attentional processes engaged by search in ASD.

## Introduction

We conducted five visual search experiments to test whether face processing abnormalities or disrupted visual attention to faces and the eye region may explain prior findings of reduced fixation of faces and eyes in autism during free viewing of scenes. Autistic Spectrum Disorders (ASDs) are prevalent [1] severe neuropsychiatric disorders that affect social relatedness, communication, and cause restricted interests and repetitive behaviors [2]. Impaired use of eye-to-eye gaze is part of one DSM-IV-TR criterion for autism, and abnormalities in gaze provide early – if not the earliest – diagnostic clues [3]. It is, therefore, important to understand the mechanics of deficient gaze to faces and eyes, given demonstrated benefits of intervening at the youngest age [4]. Further elucidating the psychological and brain-based causes of social-communicative gaze abnormalities in ASD might lead to more sensitive early diagnostic assessments.

Eye-tracking studies have shown decreased viewing of eyes and relatively increased viewing of mouths in ASD when compared to control subjects [5]. High-risk infant sibling work has shown diverging curves between six and twelve months for measures of gaze to faces in infants who do (decreasing amounts) and do not (stable to increasing amounts) develop an ASD [6]. The amount of decreased gaze to eyes also correlates with symptom severity in autistic toddlers [7]. The mechanism(s) explaining these real world and experimental findings remain(s) elusive. Current hypotheses include: attraction to audio-visual contingencies generated by mouth motion and speech sounds in ASD [8]; aversive affective valence for eyes (e.g., [9]); disrupted visual attentional “capture” for the eye region (e.g., [10]); altered high-level visual representations of faces in ASD (e.g., [11]) such that faces are not detected accurately and efficiently in search. The reported visual search experiments, therefore, address the last two hypotheses regarding attention to the eye region of the face and aspects of high-level visual processing of faces. Findings from this approach would not rule out roles for other contributing mechanisms. However, disrupted attention to and/or high-level processing of eyes in the context of faces would advance our understanding of real-world gaze-related abnormalities in ASD.

We note that not all eye-tracking studies have found that subjects with ASD spend less time fixating faces and the eye-region when compared to typical subjects (e.g., [12], [13]). Even here, Freeth and colleagues [12] found faster timecourses for initial fixation of the face in typical participants, and van der Geest and colleagues [13] reported that subjects with ASD were not sensitive to manipulation of face orientation, as compared to the typical subjects. Despite these studies’ general findings of gross equivalence of fixation across groups, the subtle differences provide further support for our strategy of employing controlled visual search experiments that include high-level manipulations of faces.

Visual search experiments have studied the cognitive operations supporting important aspects of attention to specific stimuli under various conditions [14]–[16]. More recently, some investigators have re-explored processes imputed in visual search for faces (e.g., [17]–[21]). Despite recent experiments involving emotional [22] and gaze direction [23] search targets in ASD, we are not aware of studies directly contrasting search accuracy and efficiency for face features versus objects in ASD using the methods of Treisman et al. We [24] and others (e.g., [25]; but see [26]) have recently shown intact attentional *redirection* for eye gaze in ASD children performing covert orienting tasks. Some investigators have previously reported superior search for non-face stimuli in ASD [27]–[29], although recent findings challenge this conclusion (e.g., [30], [31]). Our controlled study with a large, well-characterized sample may provide important information about this debate. Could *search accuracy or efficiency* for faces and/or eyes be impaired in ASD, despite intact attentional redirection for gaze and non-gaze stimuli? Or could perceptual abnormalities related to the high-level representation of faces in ASD limit the information that can be accessed by brain systems controlling search efficiency [32], [33]?

Studies of face perception strongly suggest that the ability to perceive faces relates to “holistic” [34] and “configural” processing [35]. “Holistic” processing refers to processing the face as a whole, while “configural” processing involves processing either the arrangement (termed 1^st^ order configural: e.g., [36]) or spacing of face features (2^nd^ order configural: e.g., [37], [38]). In most of this paper we will use the term “high-level” in place of “holistic.” Here, and in many other published papers, the true degree of non-additivity (whole being more than the sum of parts) implied by the term “holistic” is neither explicitly nor exhaustively tested. Existing operational definitions also do not allow a firm boundary to be drawn between “holistic” and “configural” effects. However, we expect that some readers might prefer to substitute “holistic” for “high-level,” throughout.

If disrupted attention to the eye region of the face or altered incorporation of eyes into high-level percepts of faces caused impaired search accuracy or efficiency under highly controlled conditions, affected individuals might manifest a variety of gaze-related impairments that are characteristically seen in autism: poor eye-to-eye gaze, impaired gaze following, and disrupted joint attention. Here, visual search experiments, involving basic and face stimuli contrasted children with and without ASD on measures of feature search, conjunction search, face in non-face-object search, and searches for eye and mouth regions. In distinction from many earlier autism visual search studies, we used sparse, circular, time-limited search arrays and required and monitored eye fixation – choices which may help isolate attentional aspects of visual search. By instructing fixation we *attempted* (but did not completely succeed; see below) to get a cleaner measure of attentional demand per item, free from the additional effects of eye movements on reaction time [39] (and see: [24]). By using uncluttered displays, we may have reduced the contribution from potential differences that ASD children may show in terms of their abilities to process items amidst clutter (see discussion).

## Methods

### Subjects and Assessments

We recruited subjects (see “Acknowledgments”) and performed studies according to approved protocols (Washington University School of Medicine’s Human Research Protection Office: HRPO). We obtained written consent from the guardians on behalf of the minors/children participants. Subjects were 9–12 year-old children. This was the second of two studies (please see [24]) involving a sample of 9–12 year-old children. The age range was determined pragmatically. Our pilot experiments revealed less than 50% success in task completion for children under nine. Pre-screening involved a brief medical history, pedigree, and demographics. We excluded subjects with a history of focal neurological deficit, strabismus, or vision that would not correct to normal acuity with glasses. Typical subjects had no first-degree relatives with an ASD or Attention-Deficit/Hyperactivity Disorder (ADHD).

We assessed each subject with: vocabulary and block design subscales (only, because of their reliability and to minimize subject burden) from the Wechsler Intellgience Scale for Children-Fourth Edition (WISC-IV: [40]), the Strengths and Weaknesses of ADHD-symptoms and Normal-behavior scale (SWAN: [41]), the Social Responsiveness Scale (SRS: [42]), Handedness Inventory [43], the Child Behavior Checklist (CBCL: [44]), and a brief neurological exam (performed by the 1st author). The DSM-IV-TR does not allow co-diagnosis of ASD and ADHD. ADHD *symptoms* were measured with both the CBCL and the SWAN scale. There were no correlations between our dimensional measures of ADHD symptoms and performance in the tasks. Children were not tested for color-blindness; green-blue color blindness is relatively rare [45], hence our choice of colors; all subjects reported being able to discriminate the targets; and color search performance was excellent (see below). Typical subjects were required to have all CBCL t-scores <60. ASD participants had (1) community MD or PhD clinical diagnoses of Autistic Disorder, Asperger’s Disorder, or Pervasive Developmental Disorder Not Otherwise Specified (PDD-NOS), (2) research ASD diagnoses as determined in our study with the Autism Diagnostic Observation Schedule (ADOS: [46]) and/or Autism Diagnostic Interview Revised (ADI-R: [47]), and (3) passed author diagnostic review (child psychiatrists JRP and/or JNC, who viewed available ADOS videos), which excluded three ASD children who met ADOS/ADI-R algorithmic inclusion. In other words, included ASD subjects were identified ASD patients who scored ADOS and/or ADI-R positive and passed MD tape review where tapes were available.

Sixty-nine children with block design and vocabulary scaled scores >6 met the above criteria and completed all search experiments. Thirty-two children (14 typical, 18 ASD) participated in our covert orienting of visual attention study [24]. Out of concern for potential effects of age, vocabulary, and block design on reaction time (RT), we were able to match (no significant difference at the group level: p>.1) 60 of the included 69 children – who showed good performance on color, eyes-absent, face, and mouth-absent – on these three measures by eliminating the nine typical children with the highest vocabulary scaled scores (>14). This strategy left: 29 (three female) typical and 31 (four female) ASD children.

In the conjunction experiment, we removed four of these 69 subjects (one typical and three ASD) from analyses because they simply pushed one response button for nearly the entire experiment (despite performing as instructed in the other four experiments). We could match 55 of these 65 children on age, vocabulary, and block design by eliminating seven typical children with the highest vocabulary scaled scores (>15) and three ASD children with the highest block design scaled scores (>17). Here we had: 30 typical (four female) and 25 (three female) ASD children (for conjunction). Summaries of the final participants’ characteristics and assessments can be seen in Tables 1, 2, 3, 4, 5, 6, 7, and 8.

**Table 1:** Demographics for Color, Face, Mouth-Absent, and Eyes-Absent.

		ASD	Typical	Total
Gender	*Male*	27	26	53
	*Female*	4	3	7
Race	*Caucasian*	30	24	54
	*African American*	1	5	6
	*Multi-racial*	0	0	0
Psychotropic Medications	*Yes*	15	0	15
	*No*	16	29	45
Vision	*Normal*	27	22	49
	*Corrected-Normal*	4	7	11
Handedness (self-report)	*Right*	26	21	47
	*Left*	3	7	10
	*Ambidextrous*	1	0	1
	*Not Reported*	1	1	2

(ASD N = 31; Typical N = 29).

**Table 2:** Clinical Information for Color, Face, Mouth-Absent, and Eyes-Absent.

		ASD Mean	ASD SD	Typ. Mean	Typ. SD	df	t	p
Age		10.9	1.3	10.9	1.1	58	−.011	0.991
WISC	*Block Scaled*	12.7	3.2	11.5	2.4	55.5	−1.66	0.103
	*Vocab Scaled*	10.2	2.8	11.0	1.4	45.1	1.48	0.147
SRS	*Total*	99.0	23.3	19.5	12.6	46.7	−16.58	0.000
SWAN*	*Total ADHD*	−16.4	15.9	10.1	16.6	57	6.27	0.000
	*Inattentive*	−7.7	9.8	4.5	8.4	57	5.13	0.000
	*Hyper-Impulsive*	−8.8	7.6	5.7	9.0	57	6.69	0.000
CBCL	*Anx/Dep*	64.1	12.1	50.8	2.0	30.7	−5.93	0.000
	*Wdrwn/Dep*	64.0	9.8	51.7	2.8	33.8	−6.61	0.000
	*Somatic*	59.7	8.7	51.8	2.6	34.2	−4.78	0.000
	*Social*	65.2	10.5	51.6	2.8	33.1	−6.86	0.000
	*Thought*	69.4	7.9	51.5	2.3	33.9	−11.97	0.000
	*Attention*	67.1	9.5	50.8	1.3	30.2	−9.32	0.000
	*Rule Breaking*	55.3	6.6	51.1	1.9	33.9	−3.38	0.002
	*Aggressive*	62.0	10.5	51.8	3.0	34.0	−5.11	0.000

(Age, WISC, SRS, N = 60; Swan, CBCL, N = 59).

* Positive SWAN scores indicate superior attention and negative scores reflect ADHD symptoms.

**Table 3:** Additional Clinical Information for ASD Participants for Color, Face, Mouth-Absent, and Eyes-Absent.

		N	Mean	SD
ADOS	*Communication* cutoff = 3(Aut),2(ASD)	31	3.3	1.8
	*Social Interaction* cutoff = 6(Aut),4(ASD)	31	6.4	2.3
	*Comm + Social* cutoff = 10(Aut),7(ASD)	31	9.5	3.4
	*Stereotypy/Restricted*	31	1.7	1.9
ADI-R	*Social Interaction* cutoff = 10	30	18.8	5.3
	*Communication* cutoff = 8	30	15.7	4.8
	*Restricted/Repet/Stereotypy* cutoff = 3	30	7.1	2.4
	*Abnormal Dev before 36 mn* cutoff = 1	30	3.4	1.5
Community Clinical Diagnosis	*Autistic Disorder*	14		
	*Asperger’s Disorder*	9		
	*PDD-NOS*	4		
	*“Autism Spectrum Disorder”*	4		

**Table 4:** ADI-R * ADOS Classification Cross Tabulation.

	ADOS Classification	No Dx	Autism Dx	Autism Spectrum Dx	Total
ADI-R Diagnosis	No Dx	0	0	0	0
	Autism Dx	1	10	19	30
	Missing	0	1	0	1
	Total	1	11	19	31

**Table 5:** Demographics for Conjunction.

		ASD	Typical	Total
Gender	*Male*	22	26	48
	*Female*	3	4	7
Race	*Caucasian*	24	25	49
	*African American*	1	5	6
	*Multi-racial*	0	0	0
Psychotropic Medications	*Yes*	13	0	13
	*No*	12	30	42
Vision	*Normal*	22	23	45
	*Corrected-Normal*	3	7	10
Handedness (self-report)	*Right*	20	22	42
	*Left*	3	7	10
	*Ambidextrous*	1	0	1
	*Not Reported*	1	1	2

(ASD N = 25; Typical N = 30).

**Table 6:** Clinical Information for Conjunction.

		ASD Mean	ASD SD	Typ. Mean	Typ. SD	df	t	p
Age		10.9	1.3	10.9	1.1	53	0.05	0.957
WISC	*Block Scaled*	12.3	2.8	11.3	2.0	42.5	−1.43	0.161
	*Vocab Scaled*	10.3	2.9	11.2	1.6	35.2	1.37	0.179
SRS	*Total*	97.9	22.4	18.8	12.7	36.5	−15.66	0.000
SWAN*	*Total ADHD*	−18.1	15.3	11.5	16.8	52	6.70	0.000
	*Inattentive*	−9.0	9.0	5.0	8.4	52	5.90	0.000
	*Hyper-Impulsive*	−9.3	7.9	6.6	9.2	52	6.66	0.000
CBCL	*Anx/Dep*	65.5	12.3	51.1	2.6	24.7	−5.6	0.000
	*Wdrwn/Dep*	64.5	9.9	51.5	2.7	25.8	−6.3	0.000
	*Somatic*	59.9	9.0	51.7	2.5	25.9	−4.4	0.000
	*Social*	65.8	9.7	51.5	2.7	25.9	−7.0	0.000
	*Thought*	70.3	7.5	51.4	2.2	26.3	−11.9	0.000
	*Attention*	68.8	9.1	50.8	1.3	23.8	−9.6	0.000
	*Rule Breaking*	56.0	7.1	51.1	1.9	25.5	−3.3	0.003
	*Aggressive*	63.6	10.6	51.7	3.0	25.9	−5.3	0.000

(Age, WISC, SRS, N = 55; SWAN, CBCL, N = 54).

* Positive SWAN scores indicate superior attention and negative scores reflect ADHD symptoms.

**Table 7:** Additional Clinical Information for ASD Participants for Conjunction.

		N	Mean	SD
ADOS	*Communication* cutoff = 3(Aut),2(ASD)	25	2.96	1.59
	*Social Interaction* cutoff = 6(Aut),4(ASD)	25	6.4	2.33
	*Comm + Social* cutoff = 10(Aut), 7(ASD)	25	9.4	3.39
	*Stereotypy/Restricted*	25	1.6	2.08
ADI-R	*Social Interaction* cutoff = 10	24	18.5	5.40
	*Communication* cutoff = 8	24	15.4	5.20
	*Restricted/Repet/Stereotypy* cutoff = 3	24	7.0	2.18
	*Abnormal Dev before 36 mn* cutoff = 1	24	3.4	1.56
Community Clinical Diagnosis	*Autistic Disorder*	10		
	*Asperger’s Disorder*	7		
	*PDD-NOS*	4		
	*“Autism Spectrum Disorder”*	4		

**Table 8:** ADI-R * ADOS Classification Cross Tabulation.

	ADOS Classification	No Dx	Autism Dx	Autism Spectrum Dx	Total
ADI-R Diagnosis	No Dx	0	0	0	0
	Autism Dx	1	8	15	24
	Missing	0	1	0	1
	Total	1	9	15	25

### Equipment and General Procedures

Participants sat in a dark room, 70 centimeters away from the stimulus display. A chin rest with a head strap minimized head movement. We presented stimuli on a 17″ CRT monitor (at 800×600 pixels, 75 Hz) controlled by a Macintosh computer running PsyScope 1.2.2 [48] and recorded responses with a PsyScope Button Box (New Micros, Inc.; Dallas, TX; 1 ms timing resolution). A separate Macintosh running PowerLab (ADInstruments, http://www.adinstruments.com) synchronized with the experiment control computer and acquired horizontal electrooculography (EOG), which provided a crude measure of central fixation (1–2 degrees resolution). Because our concern was simply monitoring fixation, we used horizontal EOG, only. A custom program automatically flagged trials involving detectable EOG deviations from central fixation. Below, percent of trials fixated is the percent of trials where no measurable horizontal deviation from the fixation mark occurred prior to button press. Vertical eye movements would not be detected well, and only the horizontal component of oblique eye movements would be seen. The likely number of eye movements was, therefore, potentially greater than the percent of trials “not fixated.”

### Experiments and Stimuli

**Figure 1:**
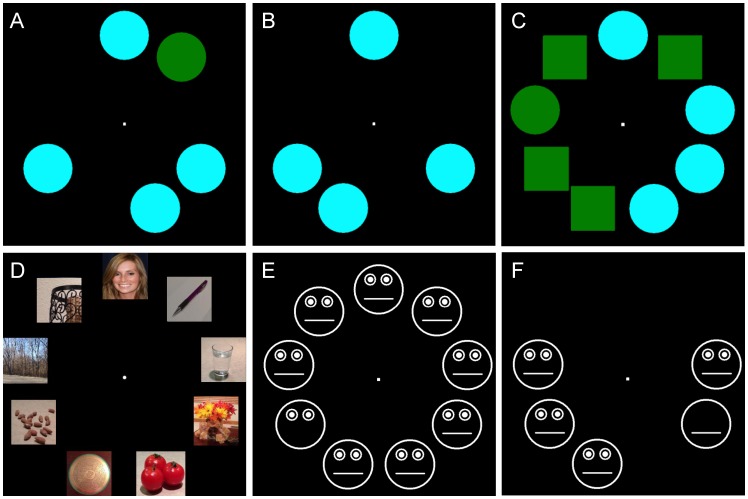
Example stimulus arrays. *A.* Five-item, target-present, color search trial display. *B.* Four-item, target-absent, color search array. *C.* Nine-item, target-present, color-shape conjunction trial. The target is the left-most circle. *D.* Target-present face search. *E.* Mouth-absent experiment target-present search. *F.* Eyes-absent experiment target-present array.

Each child attempted five different visual search experiments – color, conjunction, face, mouth-absent, and eyes-absent – in pseudorandom, counterbalanced order (Figure 1). Each experiment contained 60 trials (30 target-present and 30 target-absent trials, with 10 presentations of each set size: 3, 5, 9 items). Target-presence/absence and array size varied pseudo-randomly in a counter-balanced fashion on a trial-wise basis. Participants indicated the presence or absence of the instructed target for each experiment by pushing one of two buttons on the button box.

Both groups of children completed the battery of experiments in approximately one hour with a short break between each experiment. The instructions were identical, and the protocol was administered the same way for both groups of children. Children read the instructions on the screen before each experiment. The research assistant also read these out loud to the children. Color: “In the following task, please keep your eyes on the dot in the center of the screen, and don’t look anywhere else. On each trial you will see a different number of colored shapes. These shapes may disappear after a short while. Press the red button if there was a green circle, and press the yellow button if there wasn’t. Respond as quickly but as carefully as you can. Press any button to continue.” Conjunction: “In the following task, please keep your eyes on the dot in the center of the screen, and don’t look anywhere else. On each trial you will see a different number of colored shapes. These shapes may disappear after a short while. Press the red button if there was a green circle, and press the yellow button if there wasn’t. Respond as quickly but as carefully as you can. Press any button to continue.” Face: “In the following task, please keep your eyes on the dot in the center of the screen, and don’t look anywhere else. On each trial, you will see a different number of pictures. These pictures may disappear after a short while. Press the red button if there was a face, and press the yellow button if there wasn’t. Respond as quickly but as carefully as you can. Press any button to continue.” Mouth-absent: “In the following task, please keep your eyes on the dot in the center of the screen, and don’t look anywhere else. On each trial you will see a group of faces. The faces may disappear after a short while. Press the red button if one of the faces is missing a mouth, and press the yellow button if all of the faces have mouths. Respond as quickly but as carefully as you can. Press any button to continue.” Eyes-absent: “In the following task, please keep your eyes on the dot in the center of the screen, and don’t look anywhere else. On each trial you will see a group of faces. The faces may disappear after a short while. Press the red button if one of the faces is missing eyes, and press the yellow button if all of the faces have eyes. Respond as quickly but as carefully as you can. Press any button to continue.”

Stimuli (item size = ∼1.6 visual degrees) appeared simultaneously around an imaginary ring with a radius of 3.5 degrees from central fixation (i.e., all stimuli at equal eccentricity), while the subject fixated a point in the center of the black screen. We did not use larger stimuli because larger stimuli would not fit into 9 element arrays at this eccentricity without overlapping. Though slightly smaller than stimuli some other groups have used in face search with ASD individuals (see [23]), our basic face search findings compare to other reports [17], and same-sized face stimuli produced robust redirections of visual-spatial attention in response to lateral shifts in pupils during a recent gaze cueing covert orienting study [24], attesting to the validity of our stimuli. Each stimulus could appear pseudo-randomly at nine possible locations around the imaginary ring. Spatial patterns for target-present and target-absent conditions were counterbalanced across trials. Each trial’s array of 3, 5, or 9 stimuli were displayed for only 1500 ms to reduce the tendency to make eye movements (a shorter display time might have been more ideal, but our pilot experiments showed unacceptably low accuracy on our experiments when the display time was shortened for these children). Then, a blank screen with fixation mark remained until the child responded. We jittered the inter-trial interval (minimum = 1100 ms + random delay = 1000, 1500, or 2000 ms) to prevent anticipation of trial onset.

Color search involved detecting a green circle amidst cyan circle distracters. In conjunction search, children looked for a green circle amidst green square and cyan circle distracters. The face experiment involved detecting faces in displays containing non-face objects and scenes from the Microsoft Photo Gallery (http://dgl.microsoft.com) (as used in: [17]; the faces were not selected to have any specific gaze direction). For copyright purposes, Figure 1D is an illustrative example, created ourselves, of a 9-item face target-present stimulus array, as used in the experiment (note: anonymous peer reviewers were able to view actual stimulus examples). Search experiments, including many seminal studies, do not typically involve tests using isoluminant stimuli [15], [17], [22], [27]–[29]; color stimuli (see Figures 1A–D), here, were not luminance-equated. Mouth-absent search involved detecting a cartoon face with a missing mouth in arrays of full cartoon faces. The eyes-absent search experiment required children to find a cartoon face with missing eyes. The mean luminance of the mouth-absent and eyes-absent faces in Figure 1E and 1F would obviously be lower than the full faces, which by definition had more white pixels. We feel confident that the *between-group* differences in the eyes-absent experiment results reported below did not result from a failure to equate the luminance of the stimuli.

Line drawn cartoons (see Figure 1) were used to enhance stimulus control, reduce the chance of inadvertently introducing low-level stimulus confounds, and allow for ease in generating eye and mouth manipulations. Line drawn cartoon stimuli have produced robust neuroimaging findings in studies of visual attention to eye-gaze shifts, while maximizing stimulus control (e.g., [49]), and others have used cartoon stimuli in ASD visual search experiments (e.g.: demonstration of intact threat detection in Asperger’s disorder using search [22]).

The logic for our two feature-absent target searches was two-fold: 1) Detecting eyes in arrays that contained faces with missing eyes would be trivial (akin to finding a Q in an array of O’s [15]: efficient, feature-present search). 2) Removing eyes and mouths provided contrasting high-level eyes and mouth conditions for examining degrees to which detecting the absence of these features is modulated by the presence of other key facial features, across ASD and typical children (analogously to studies that have used “part/whole” manipulations to probe for contextual influences of the entire face on its component features [50]).

### Design and Analyses

These experiments involved mixed model multi-factorial designs. The between-subjects variable was diagnosis (ASD, typical). Within-subjects variables included target (present, absent) and array (3, 5, 9 items). Accuracy (percent correct) and condition median RT for correct trials were key dependent variables, analyzed separately. We used the Statistical Package for the Social Sciences (SPSS) 16.0 and 18.0 (SPSS, Inc.) for repeated measures analysis of variance (ANOVA), T-Tests, bivariate correlation, and linear regression. For repeated measures ANOVAs where sphericity was violated, Greenhouse-Geisser corrections were applied. When necessary for t-tests, degrees of freedom corrections were reported to account for unequal variances between groups. In the primary accuracy and RT analyses, we consider p = .01 to be significant to correct alpha for the five experiments but will report uncorrected p-values for the reader to draw his/her own conclusions. EOG data was not of acceptable quality in all children; therefore, fewer degrees of freedom are reported for EOG analyses compared with accuracy and RT. We did not conduct cross-experiment ANOVAs, as this practice is discouraged for search experiments (see [16]), given the manner in which stimulus similarity/dissimilarity may modulate search RT functions and the challenge of equating similarity across experiments, such as for our conjunction and color experiments.

### Key Effects of Interest

We explored potential main effects of diagnosis and interactions between diagnosis, target, and/or array size on accuracy and median RT. For feature searches that are efficient, RTs do not increase with array size (the target feature “pops-out,” no matter how many stimuli are in the display) [15]. The color search experiment is a simple feature search task (efficient search control condition). Both groups of children were expected to show high accuracy and shallow or flat RT profiles. Thus, we predicted no significant main effect of array on RT or accuracy in the color experiment, and no interactions with diagnosis. Conjunction search is typically inefficient with RT often increasing in a linear manner with the number of display items. Here, we expected to see this pattern in the typical children, with a potentially similar effect on accuracy. However, based on the ASD conjunction search literature findings described in the introduction, we expected to see either globally faster and/or more accurate responses in the ASD children (main effect of diagnosis), or more shallow RT and/or accuracy versus array size slopes (diagnosis * factor interaction). Face in non-face-object search studies have reported shallow (sometimes flat – see, e.g., [17]) slopes on target-present trials and steeper slopes on target absent trials. This pattern (i.e., efficient target-present but inefficient target absent search) would be reflected in a significant target * array interaction on RT. The cited face search literature pertains to typical adult subjects, only. We are not aware of published data on face in non-face object search in children, typical or with ASD. Here, we hypothesized that the typical children would show the reported adult RT pattern but that the children with ASD would show less efficient target-present search (a diagnosis * target * array size interaction) and potentially reduced accuracy. As stated, our eyes- and mouth-absent experiments require the children to search for the *feature-absent* stimulus. A feature-absent stimulus should *not* produce flat target-present RT slopes [15]. Relatively flat (feature-absent) target-present RT slopes might, therefore, result from a contrast set up by the high-level arrangement of features in the target versus non-target stimuli. Non-target RT slopes would be steeper because no such high-level contrast exists. Pilot test results from our lab confirmed the presence of flat target-present RT slopes for target-present eyes-absent, but not mouth-absent, stimuli in typical adults. We, therefore, predicted the typical children would show flat target-present and steep target absent RT slopes in the eyes-absent experiment. We predicted potentially steep RT slopes for target-present and target absent conditions in the mouth-absent experiment. Due to prior reports of poor eye region fixation in ASD, we predicted the children with ASD would show steeper target-present slopes (compared to the typical children) for the eyes-absent experiment with reduced accuracy. Based on prior report of relatively increased viewing of mouths in ASD, we predicted the children with ASD might show enhanced RT and accuracy profiles, as compared to the typical children.

Our study did not involve explicit empirical tests of theories that we believe may be potentially informative about face in non-face-object search [19], [51]. Our goal was to see whether search experiments with different targets might dissociate children with and without ASD to inform future behavioral and imaging work, which may clarify mechanism further. Therefore, to maintain a theory-neutral a stance, we will describe RT profiles with relatively flat target-present and steep target-absent slopes in terms of the statistical significance of target * array size interactions.

## Results

We found no compelling reason to exclude any children because of speed-accuracy trade-offs. There were multiple significant effects; we will report statistics that relate in the most salient ways to our central questions.

### Color

**Figure 2:**
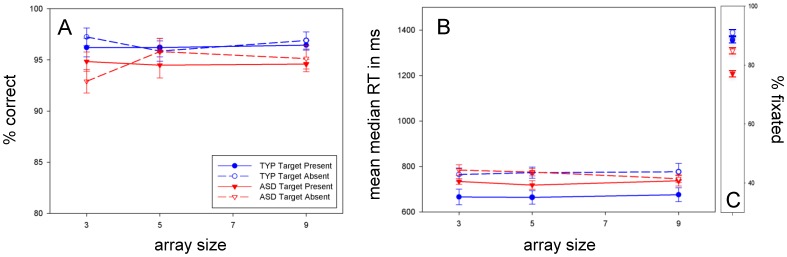
Color search. *A:* Accuracy (% correct) versus array size. *B:* RT versus array size; Color search was accurate and efficient for both groups. *C:* Percent of trials fixated, measured with horizontal EOG. ASD children trended for poorer fixation. All plotted Standard Error of the Means (SEMs) are adjusted for repeated measures (error bar = 1 adjusted SEM).

**Accuracy:** As depicted in Figure 2A, ASD children were not significantly less accurate than typical children [F(1, 58) = 2.066; p = .156; ŋ_p_^2^ = .034 ]. **RT:** Likewise, Figure 2B shows that ASD children were not significantly [F(1, 58) = .283; p = .596; ŋ_p_^2^ = .005] slower than typical children. Analyses revealed a trend for target [F(1, 58) = 6.140; p = .016; ŋ_p_^2^ = .096] (again, p = 0.01 reflects the critical level because of the five experiments), but no effect of array size [F(1.779, 103.181) = .060; p = .925; ŋ_p_^2^ = .001] or diagnosis * array size [F(1.779, 103.181) = .555; p = .556; ŋ_p_^2^ = .009] – indicating efficient search for both groups. **Fixation:** In Figure 2C, typical children appeared to fixate better than ASD children. A 2 (diagnosis: typical, ASD)×2 (target: present, absent) mixed-model, repeated measures ANOVA on percent of trials fixated showed that this difference reached trend level [F(1, 49) = 5.326; p = .025; ŋ_p_^2^ = .098].

### Conjunction

**Figure 3:**
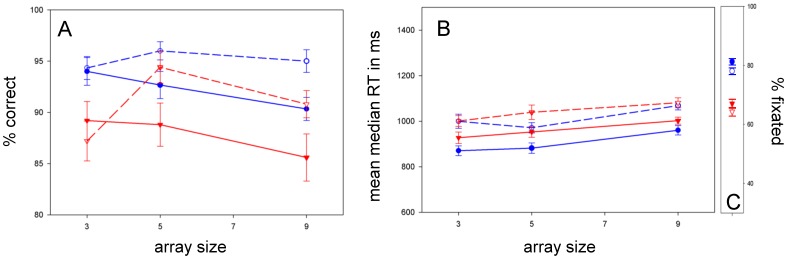
Color-shape conjunction search. *A:* ASD children were *not* more accurate at conjunction search. *B:* RTs increased with array size for target-present and target-absent searches in both groups. ASD children were *not* faster at conjunction search. *C:* ASD children trended for poorer fixation.

**Accuracy:** The difference in accuracy between ASD and typical children shown in Figure 3A did not reach significance [F(1, 53) = 3.571; p = .064; ŋ_p_^2^ = .063]. ANOVA revealed a main effect of target [F(1, 53) = 8.012; p = .007; ŋ_p_^2^ = .131] and a target * array size trend [F(1.760, 93.278) = 4.387; p = .019; ŋ_p_^2^ = .076]. **RT:** In Figure 3B, ASD children’s RTs were not significantly different from the typical children’s [F(1, 53) = .189; p = .665; ŋ_p_^2^ = .004]. RT ANOVA showed main effects of target [F(1, 53) = 26.877; p = .000; ŋ_p_^2^ = .336] and array size [F(2, 106) = 10.327; p = .000; ŋ_p_^2^ = .163] with neither target * array size [F(2, 106) = .087; p = .917; ŋ_p_^2^ = .002] nor diagnosis * array size interactions [F(2, 106) = .816; p = .445; ŋ_p_^2^ = .015] – implying serial target present and absent slopes that did not differ across groups. **Fixation:** Figure 3C shows that ASD children showed a trend for worse fixation (repeated measures ANOVA on percent of trials fixated: [F(1, 48) = 6.289; p = .016; ŋ_p_^2^ = .116]).

### Face

**Figure 4:**
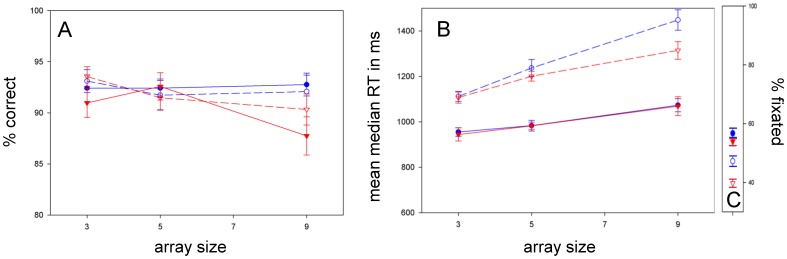
Face in non-face-object search. *A:* Contrary to our prediction, both groups accurately searched for faces in arrays of non-face objects. *B:* Both groups showed shallow target-present/steep target-absent RT profiles. ASD children were not slower than typical children. *C:* Both groups evidenced poor central fixation.

**Accuracy:** Figure 4A shows that ASD and typical children were similar in accuracy [F(1, 58) = .238; p = .628; ŋ_p_^2^ = .004]. **RT:** In Figure 4B ASD children and typical children also had similar RTs [F(1, 58) = .145; p = .705; ŋ_p_^2^ = .002]. There were main effects of target [F(1, 58) = 123.479; p = .000; ŋ_p_^2^ = .680] and array size [F(2, 116) = 32.768; p = .000; ŋ_p_^2^ = .361] and a target * array size interaction [F(1.503, 87.168) = 7.102; p = .003; ŋ_p_^2^ = .109] with no diagnosis * target * array size interaction [F(1.503, 87.168) = 1.870; p = .169; ŋ_p_^2^ = .031] – demonstrating similar RT profiles for faces in *both* groups. These findings ran also ran contrary to our predictions. **Fixation:** In Figure 4C ASD and typical children did not differ significantly in fixation [F(1, 49) = .700; p = .407; ŋ_p_^2^ = .014], which was poor.

### Mouth-absent

**Figure 5:**
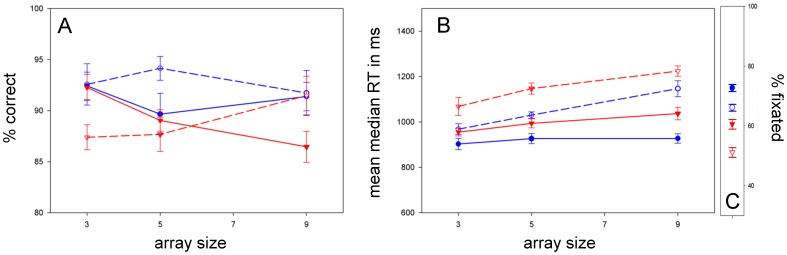
Mouth-absent search. There was no ASD advantage for mouth region search, as measured by *A:* Accuracy. *B:* RT. Both groups showed shallow target-present/steep target-absent RT profiles. *C:* Fixation was poor, with a trend for worse fixation in ASD.

**Accuracy:** Figure 5A shows accuracy profiles for ASD and typical children that were not significantly different [F(1, 58) = 1.430; p = .237; ŋ_p_^2^ = .024]. **RT:** ASD children were not significantly [F(1, 58) = 2.348; p = .131; ŋ_p_^2^ = .039] slower than typical children in Figure 5B. There were main effects of target [F(1, 58) = 52.963; p = .000; ŋ_p_^2^ = .477] and array size [F(2, 116) = 16.828; p = .000; ŋ_p_^2^ = .225] on RT and a significant target * array size interaction [F(2, 116) = 5.005; p = .008; ŋ_p_^2^ = .079] (similar to the pattern for face targets, as above). There was no significant diagnosis * target * array size interaction [F(2, 116) = .818; p = .444; ŋ_p_^2^ = .014]. **Fixation:** Figure 5C shows that typical children trended for better fixation [F(1, 49) = 5.583; p = .022; ŋ_p_^2^ = .102].

### Eyes-absent

**Figure 6:**
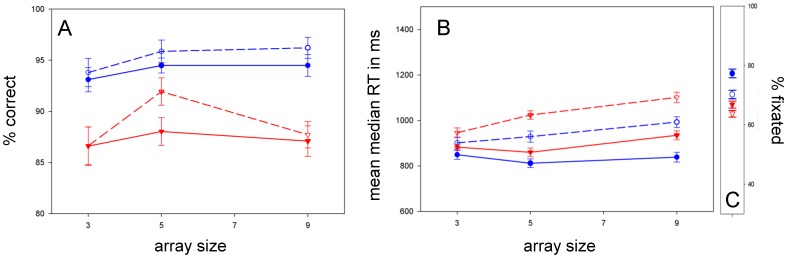
Eyes-absent search. *A:* Eye region search accuracy was significantly worse in ASD. *B:* However, both groups showed similar, shallow target-present/steep target-absent RT functions. *C:* Fixation was marginally better and not different across groups.

**Accuracy:** Figure 6A shows that ASD children were less accurate than typical children [F(1, 58) = 8.492; p = .005; ŋ_p_^2^ = .128]. **RT:** The cross-group RT difference in Figure 6B did not reach significance [F(1, 58) = 1.332; p = .253; ŋ_p_^2^ = .022]. ANOVA revealed main effects of target [F(1, 58) = 49.276; p = .000; ŋ_p_^2^ = .459] and array size [F(1.798, 104.262) = 13.695; p = .000; ŋ_p_^2^ = .191] and a target * array size interaction [F(2, 116) = 6.960; p = .001; ŋ_p_^2^ = .107] but no diagnosis * target * array size interaction [F(2, 116) = .234; p = .791; ŋ_p_^2^ = .004]. **Fixation:** In Figure 6C fixation was not significantly worse for ASD children [F(1, 49) = 2.431; p = .125; ŋ_p_^2^ = .047].

### Comparison of Matched and Unmatched Groups

Cross-group matching in ASD research is particularly complex because of the mixed neuropsychological profiles commonly seen in ASD sub-groups [52]. Different matching strategies may also be more appropriate for different kinds of research and for experiments involving different dependent variables. Before matching, RT trended faster with increasing block design score for ASD children during conjunction search [r = −.358, p = .062]. Above, we chose to match on age, vocabulary, and block design, as we did in our recent study of covert orienting in ASD and typical children [24]. Could a failure to see evidence of enhanced conjunction search in ASD have possibly resulted from our choice of matching strategy? Here, we re-ran ANOVAs for all experiments on the larger samples that were not matched on all three key measures (age, block design, and vocabulary): N = 65 for conjunction – where age did not differ, block design showed an ASD>typical trend [t(43.632) = −1.895; p = .065], and vocabulary scaled scores were lower in ASD [t(63) = 2.563; p = .013]; and N = 69 for the other four experiments – where age and block design did not differ, but vocabulary scaled scores were lower in ASD [t(67) = 3.085; p = .003]. Table 9 summarizes the above-described pattern of results in the matched samples and contrasts it with a similar summary for identical analyses performed on the larger set of task completers that did not match on all three variables. Minor differences in the global pattern of results for this full, imperfectly matched dataset include: ASD children’s conjunction search accuracy now trends (p<0.05) for poorer (not better) performance than the typical children’s. Poorer conjunction and mouth-absent fixation in ASD reaches p<0.01 level significance. Fixation during eyes-absent search in ASD trends for poorer quality (p<0.05).

**Table 9:** Patterns of results for matched and unmatched groups.

Matched	Experiment	N	%correct	RT	DX*RT	RT pattern	fixation
	Color	60	n.s.	n.s.	n.s.	Efficient	ASD<TYP*
	Conjunction	55	n.s.	n.s	n.s.	Serial	ASD<TYP*
	Face	60	n.s.	n.s.	n.s.	Interaction	n.s.
	Mouth-Absent	60	n.s.	n.s.	n.s.	Interaction	ASD<TYP*
	Eyes-Absent	60	ASD<TYP**	n.s.	n.s.	Interaction	n.s.
**Unmatched**	**Experiment**	**N**	**%** **correct**	**RT**	**DX** * **RT**	**RT pattern**	**fixation**
	Color	69	n.s.	n.s.	n.s.	Efficient	ASD<TYP*
	Conjunction	65	ASD<TYP*	n.s.	n.s.	Serial	ASD<TYP**
	Face	69	n.s.	n.s.	n.s.	Interaction	n.s.
	Mouth-Absent	69	n.s.	n.s.	n.s.	Interaction	ASD<TYP**
	Eyes-Absent	69	ASD<TYP**	n.s.	n.s.	Interaction	ASD<TYP*

DX = diagnosis; n.s. = not significant;

* p<0.05;

** p<0.01.

Some investigators might be less concerned about effects of block design on RT, might argue that elevated block design is an important part of the ASD phenotype, and question whether our failure to find enhanced conjunction search resulted from our need to eliminate high-block design ASD subjects during the culling procedure to the match groups. We, therefore, re-ran ANOVA on a subset (N = 60) that was matched (p for cross-group difference >.1) on age and vocabulary but not block design (block design showed an ASD>typical trend [t(45.408) = −1.955; p = .057]). Here, the ASD children still trended for lower (not higher) accuracy than the typical children [F(1, 58) = 4.522; p = .038; ŋ_p_^2^ = .072], with no difference (not faster) in RT [F(1, 58) = .242; p = .625; ŋ_p_^2^ = .004].

### Additional Analyses

To explore whether the cross-group difference in eyes-absent accuracy might have resulted from the children with ASD relying more on feature- or feature-conjunction-based processes, we explored correlations between accuracy for the different experiments (8 correlations: critical alpha level = .00625). Eyes-absent accuracy did correlate with conjunction accuracy in the ASD [r = .573, p = .003] but not the typical children [r = .424, p = .020]. However, simple color feature search also correlated with eyes-absent accuracy in both groups (typical [r = .530, p = .003], ASD [r = .611, p = .000]), and eyes-absent accuracy did not correlate with face search in typical children [r = .147, p = .446], while it did in the ASD children [r = .585, p = .001] despite the similarity of RT and accuracy profiles across groups during the face experiment. Finally, conjunction accuracy did correlate with face search accuracy in both groups (typical [r = .832, p = .000], ASD [r = .656, p = .000]). Finally, eyes-absent accuracy did not correlate with SRS score (for typical children [r = −.077, p = .691] nor for the children with ASD [r = −.315, p = .084]).

## Discussion

Five visual search experiments explored whether impaired visual attentional processes and/or altered representations of faces and face features may explain aspects of autism and experimental reports of reduced fixation of faces and eyes in ASD. Our sample size is relatively large for this sort of work. We matched children at the group level on block design, vocabulary, and age. We used circular (targets and distracters at equal eccentricity), time-limited stimulus displays, and instructed and monitored for central eye fixation. Comparing accuracy and RT patterns across all experiments allowed some separation of the degrees to which potential impairments in attention and face perception contributed to the performance differences that we observed for the ASD children.

There were no significant interactions between diagnosis and RT profiles in *any* of the experiments, suggesting the underlying attentional processes engaged by visual search are both intact in ASD and similar to those in the typical children, as we recently reported for attentional redirection [24]. The similar RT patterns for ASD and typical children across the five experiments extend our knowledge about visual search in ASD from basic stimuli to faces and eyes (see also: [22], [23]).

One of our most important findings is that ASD children did not show superior color-shape conjunction search. The failure to find enhanced conjunction search does not seem likely to be attributed to a lack of power, since the numerical trends were for reduced (not increased) conjunction accuracy and slower (not faster) conjunction RT in ASD. Some investigators have asserted that prior demonstrations of enhanced conjunction search in ASD reflect enhanced discriminatory (see: [30], [53]) rather than better visual attentional capabilities. More recently, investigators have suggested superior ASD search abilities may involve de-cluttering [31], greater perceptual load capacities [54], or differential eye fixation patterns [30]. Possibly, we eliminated these advantages by presenting sparse displays and instructing subjects to fixate. Our results are, indeed, consistent with those of Baldassi et al.’s (2009) sparse (also, circular arrays with central fixation) search display results: i.e., comparable performance between ASD and typical subjects. Baldassi and colleagues also showed superior performance in ASD when the target was crowded with distractors. The convergence of our result with theirs helps reconcile the apparent contradiction with earlier reports of superior conjunction search in ASD.

As we predicted, color search performance was comparable for both groups. We did not have as strong a prediction about mouth-absent search, but our results appear consistent with some reports of differential performance for eyes versus mouths in ASD being explained by deficient eye- and not superior mouth-condition performance (see, e.g., [55]). Our negative finding, here, also makes sense in light of recent report about preferential orienting to non-social audio-visual contingencies in autism [8]. In this paper Klin and colleagues argued that increased sensitivity to audio-visual contingency and decreased sensitivity to biological motion could explain past reports of preferential viewing of mouths (where movement coincides with the sounds that come out) over eyes in ASD. Possibly, with dynamic stimuli and sound, we would have observed such a preference.

Surprisingly, ASD children did not differ from typical children in either face search accuracy or RT profiles. Both groups of 9–12 year-olds were accurate and demonstrated shallow target-present and steep target absent RT slopes. Therefore, a relatively elevated preference to view objects in ASD does not appear to result from a deficit in search accuracy/efficiency for faces, *per se*. While several independent groups [17], [20], [21] have demonstrated flat target-present/steep target-absent search RT profiles for faces in typical adults, we are not aware of any published reports of such profiles for faces in typical children.

Another important result is that ASD children showed reduced accuracy for eye region search (p = .005). As above, globally intact search RT profiles imply that visual search efficiency is not disrupted for either face or non-face stimuli in ASD. Relatedly and as stated, we also recently showed that that the exogenous, endogenous, and reflexive reorienting of visuospatial attention for gaze, box, and arrow cues is intact in children with ASD [24]. Therefore, reduced accuracy for eyes-absent search in ASD children appears to result from A) selective compromise to an eye-specific aspect of attention engaged by search and/or B) high-level perceptual abnormalities. Though the former is possible, we emphasize again that processes engaged by search for whole faces and the mouth region appear intact, and presumably some of the same high-level attentional machinery would mediate search efficiency for the eye region. However, caution is warranted in comparing results from the face and eyes-absent (and mouth-absent) search experiments because the face experiment used face photographs and the eyes-absent experiment used schematic faces. Future studies may extend the validity of this comparison, in part by using real face photographs with removed mouths and eyes. The findings of the individual experiments stand, regardless of the cross-experiment interpretation.

Disruption to processes closer to sensory inputs or bottom-up/feed-forward attentional operations might explain the eyes-absent search results as follows: In typical development eyes contribute to high-level/holistic face representations. Removing eyes disrupts this arrangement, and non-holistic (eyes-absent face) in holistic (full-face distracters) search is efficient because the non-holistic-versus-holistic feature contrast brings attention to the target. No such high-level feature contrast exists in the target-absent condition. If eyes don’t contribute as strongly to the high-level/holistic representation of faces in ASD, then this effect would be diminished, reducing accuracy. This interpretation does *not* imply the complete absence of a high-level face representation in ASD. Rather, eyes do not contribute as potently to this high-level representation, hence the lack of a high-level feature contrast in ASD during eyes-absent search, which results in lower performance compared to typical children. The lack of a cross-group difference in the mouth-absent experiment may result from a similar incorporation of mouths into high-level/holistic face representations in ASD, as in typical children. Removing the mouth compromises “faceness” to a similar extent in both groups. This explanation for our eyes-absent and mouth-absent search experiment findings could account for previously reported differences in eye versus mouth viewing in ASD during eye-tracking studies or natural scene viewing (e.g. [5]).

This interpretation appears consistent with findings from a number of ASD face processing studies. Here, we highlight select reports from a relatively large body of work (for review see: [56]). Hobson et al. found that autistic adults relied more on information from the mouth and forehead when making emotional judgments [57]. Klin et al. used eye-tracking to demonstrate that autistic adolescents and adults found eyes less salient and mouths more salient than typical viewers did [5]. Joseph and Tanaka reported a whole-face advantage for mouths, only, in high-functioning ASD children during part-whole testing; their ASD children were deficient when test conditions required reliance on eyes [50]. Jones et al. showed that the degree of reduction in gaze to others’ eyes predicted social impairment [7]. Pellicano and Macrae recently described impaired eye gaze direction-specific effects on person-related judgments in ASD children, implying eyes are not incorporated into facial identity representations in ASD [58]. Wolf et al. found face recognition impairments across orientation, feature, and expression changes and impaired featural and “configural” discriminations based on the eye region, with preservation of such discriminations involving the mouth region [11]. Thus, a fair amount of evidence points to eyes adding less to the high-level representation of faces in ASD.

Our interpretation of the results as favoring high-level face perceptual disruption(s) in ASD over attentional abnormalities seems to fit with conclusions drawn by researchers who have investigated gaze and threat detection in ASD using search. Ashwin et al. [22] found intact threat detection in Asperger’s Disorder during visual searches involving cartoon faces with emotional expressions, but inverting the faces differentially affected the ASD subjects – suggesting intact basic search mechanics with altered high-level processing of faces. Senju and colleagues [23] reported intact gaze direction detection in autistic children during searches with (isolated) eyes and full faces, but face inversion did not affect the ASD children, while it degraded performance for the typical children. These results, again, imply generally intact visual search for eyes – in isolation, or as extracted features from full faces (i.e., the hypothetical feature-positive task alluded to in our methods) but altered incorporation of eyes into face representations as affected by inversion. However, it is possible that weaker eye-specific attentional processes in ASD might be obscured by presenting eyes in isolation or by tasks where attention to eyes is specifically instructed.

In secondary analyses, we explored whether patterns of accuracy across the study gave any further insights into differential use of feature-based versus high-level processes for the studied face manipulations. However, we were not able to paint a coherent picture. Our sense is that the described pattern of inter-experiment accuracy correlations may most parsimoniously approximate to “good” subjects *generally* doing better across the board, but future work is clearly needed. We also did not find any correlation between performance and SRS within the ASD or typical groups, despite the significant cross-group difference in eyes-absent accuracy.

We instructed and monitored central fixation and used time-limited displays to discourage eye movements. These measures were employed in an attempt to get a cleaner (free from additive or interactive effects of eye movements on RT) sense of attentional dwell time per display item. There were not enough trials to analyze accuracy and RT separately for fixated versus non-fixated trials. While both groups had great difficulty, the ASD children made more eye movements when trying to maintain central fixation – numerically so in all comparisons and significantly (p<0.01) so for two of five experiments in analyses on the unmatched groups. This finding is consistent with recent observations during our studies of covert visuospatial orienting [24] and may provide an explanation for the numerically increased RTs in search. We think it is likely that excess eye movements in ASD reflect problems with oculomotor control, not visuospatial attention (see discussion in: [24]). We qualify that neither our search nor our covert orienting experiments were designed to dissociate increased saccading from impaired engagement of attention at the fixation mark – these empirical results were serendipitous.

### Conclusions

By better isolating the attentional aspects of search with our design (uncluttered, time-limited displays; fixation instructed), we showed ASD children are *not* generally superior at conjunction visual search but do demonstrate similar search RT profiles for face and non-face stimuli. We propose that reduced importance of eyes for high-level face representations and/or selective compromise to an eye-specific aspect of attention engaged by search, explains (to a good approximation) real-world abnormalities in use of gaze and may constitute a mechanism for eye-tracker experiment reports of reduced viewing of eyes in ASD. These results bear on our understanding of the ontogeny of social deficits in ASD, suggest an avenue of further exploration in early detection research, and have direct clinical relevance, given recent demonstrations of improved “holistic” eye perception in ASD following intensive training [59].
